# Collecting Kinematic Data on a Ski Track with Optoelectronic Stereophotogrammetry: A Methodological Study Assessing the Feasibility of Bringing the Biomechanics Lab to the Field

**DOI:** 10.1371/journal.pone.0161757

**Published:** 2016-08-25

**Authors:** Jörg Spörri, Christian Schiefermüller, Erich Müller

**Affiliations:** Department of Sport Science and Kinesiology, University of Salzburg, Hallein-Rif, Austria; Rush University Medical Center, UNITED STATES

## Abstract

In the laboratory, optoelectronic stereophotogrammetry is one of the most commonly used motion capture systems; particularly, when position- or orientation-related analyses of human movements are intended. However, for many applied research questions, field experiments are indispensable, and it is not a priori clear whether optoelectronic stereophotogrammetric systems can be expected to perform similarly to in-lab experiments. This study aimed to assess the instrumental errors of kinematic data collected on a ski track using optoelectronic stereophotogrammetry, and to investigate the magnitudes of additional skiing-specific errors and soft tissue/suit artifacts. During a field experiment, the kinematic data of different static and dynamic tasks were captured by the use of 24 infrared-cameras. The distances between three passive markers attached to a rigid bar were stereophotogrammetrically reconstructed and, subsequently, were compared to the manufacturer-specified exact values. While at rest or skiing at low speed, the optoelectronic stereophotogrammetric system’s accuracy and precision for determining inter-marker distances were found to be comparable to those known for in-lab experiments (< 1 mm). However, when measuring a skier’s kinematics under “typical” skiing conditions (i.e., high speeds, inclined/angulated postures and moderate snow spraying), additional errors were found to occur for distances between equipment-fixed markers (total measurement errors: 2.3 ± 2.2 mm). Moreover, for distances between skin-fixed markers, such as the anterior hip markers, additional artifacts were observed (total measurement errors: 8.3 ± 7.1 mm). In summary, these values can be considered sufficient for the detection of meaningful position- or orientation-related differences in alpine skiing. However, it must be emphasized that the use of optoelectronic stereophotogrammetry on a ski track is seriously constrained by limited practical usability, small-sized capture volumes and the occurrence of extensive snow spraying (which results in marker obscuration). The latter limitation possibly might be overcome by the use of more sophisticated cluster-based marker sets.

## Introduction

In recent years, optoelectronic stereophotogrammetry has become one of the most commonly used systems for measuring human kinematics under laboratory conditions. It allows a highly accurate and precise three-dimensional (3D) reconstruction of the instantaneous position of passive or active markers with respect to a predefined global frame (i.e. systematic and random instrumental errors of below 2 mm) [1–3]. While systematic instrumental errors are mainly due to photogrammetric calibration inaccuracies and instrumental non-linearities, random instrumental errors most likely result from electronic noise, marker-flickering, and marker image shape distortion as a consequence of velocity effects or obscured markers [2].

Field experiments are indispensable for answering many biomechanical research questions that cannot be answered within controlled laboratory environments. This is particularly the case when investigating outdoor sports, such as alpine skiing, where the involvement of representative real-life settings (i.e. playground, sports facilities, climatic conditions, and so on) is essential to obtain valid results. However, collecting highly accurate kinematic data under such circumstances (e.g. on a ski track) is challenging. Additionally, to cover the entire area of interest, large capture volumes are necessary; therefore, complex multi-camera set-ups or wearable measurement systems (e.g. inertial measurement units and/or differential global navigation satellite systems) are needed [4–8].

Traditionally, systems of multiple fixed or panned/tilted/zoomed cameras (hereinafter called “video-based 3D kinematics”) have been used for the purpose of stereophotogrammetry in alpine skiing research [4, 9–17]. Since this method does not allow automatic marker tracking, substantial efforts have to be undertaken with regard to a manual digitizing process. At first glance, this problem might easily be overcome by the automatic tracking functions when using optoelectronic stereophotogrammetry during field experiments. However, it is not a priori clear whether the high standard of accuracy and precision observed under controlled laboratory conditions also can be achieved under challenging in-field conditions. For instance, when collecting kinematic data on a ski track, the effects of higher velocities and substantially obscured markers due to inclined/angulated postures or snow spraying may increase the occurrence of measurement errors. Moreover, the use of a minimally warming ski suit may change the number of soft tissue artifacts, which are known to be the most critical source of error in marker-based human movement analysis [18, 19].

More recently, new wearable measurement technologies have emerged resulting in several alternative approaches for collecting kinematic data on a ski track [5–8]. However, for some specific research questions, their major advantages (i.e. a superior practical usability and an unrestricted capture volume) must be weighed against compromises in the obtained signal’s exactness (e.g. when determining 3D positions of specific anatomical landmarks and/or orientations of human segments). Inertial measurement units, for example, cannot measure position and orientation directly. They are estimated by integrating acceleration and angular velocity data. Thus, when position- or orientation-related analyses of human movements need to be exact, optoelectronic stereophotogrammetry might provide a more direct and rational way of measurement. However, little is known about their practical usability under in-field conditions.

Therefore, the aims of the current study were to (1) assess the instrumental errors of kinematic data collected with optoelectronic stereophotogrammetry on a ski track, (2) investigate the magnitudes of additional skiing-specific sources of errors and soft tissue/suit artifacts, and (3) explore the practical usability of optoelectronic stereophotogrammetric systems for alpine skiing-related field experiments.

## Materials and Methods

### Ethics statement

This study was approved by the Ethics Committee of the Department of Sport Science and Kinesiology at the University of Salzburg (approval number: EC_NR. 2010_03). The subject described in this article gave written informed consent to participate in this study, and for his image to be published. Other permissions were not required.

### Data collection

**Fig 1:**
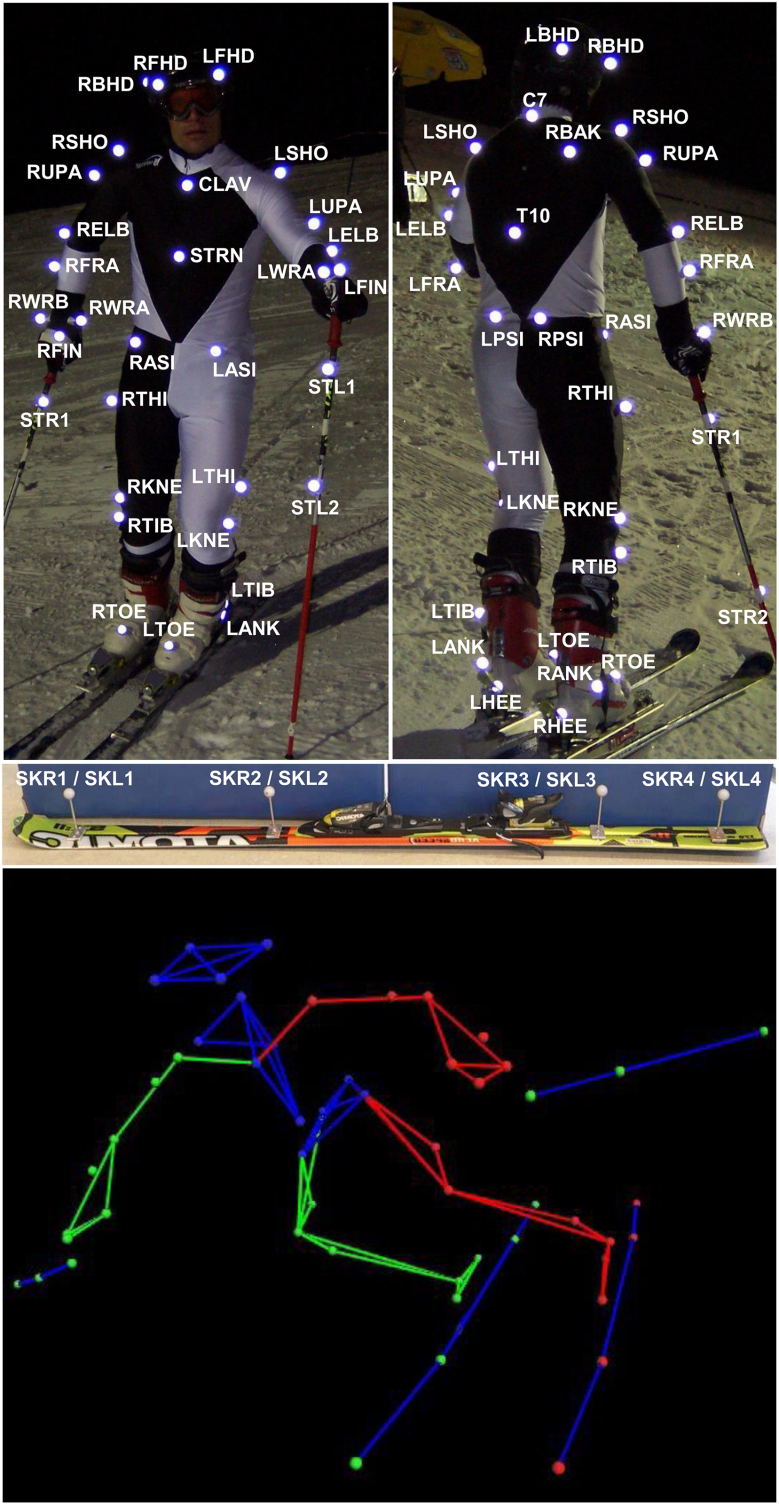
Skier equipped with the *PLUG-IN-GAIT* marker-set and additional markers on the skis and poles. Top: body and pole marker placement. Middle: equipment used with ski marker placement. Bottom: reconstructed 3D-model.

During a biomechanical field experiment on a ski track, kinematic data were collected by an optoelectronic stereophotogrammetric system (hereinafter called “optoelectronic system”). In the experiment, the following measurements were conducted: first, the 3D position data of three passive markers attached to a rigid bar (i.e. the *VICON* standard wand, *VICON* Motion Systems Ltd, UK) were captured at rest (static measurement, 1 trial). Second, the 3D position data of the same markers were also measured at motion, while being carried and lifted by a skier passing the system’s capture volume (dynamic measurement, average speed ~ 25 km/h, 1 trial). Third, the 3D kinematic data of one highly-skilled expert skier were collected while he performed a ski turn (dynamic measurement, average speed ~ 50km/h, 2 trials). The passive markers on the skier’s body were attached according to the *PLUG-IN-GAIT* model (Fig 1), and were directly fixed on the skin and pierced through a thin, highly elastic, and minimally warming ski suit. In order to avoid additional marker movements relative to the underlying bone as a consequence of wearing a ski suit, small holes around the markers were cut into the suit. The model’s foot markers were mounted on the ski boots, and additional markers were attached to the skis and ski poles.

**Fig 2:**
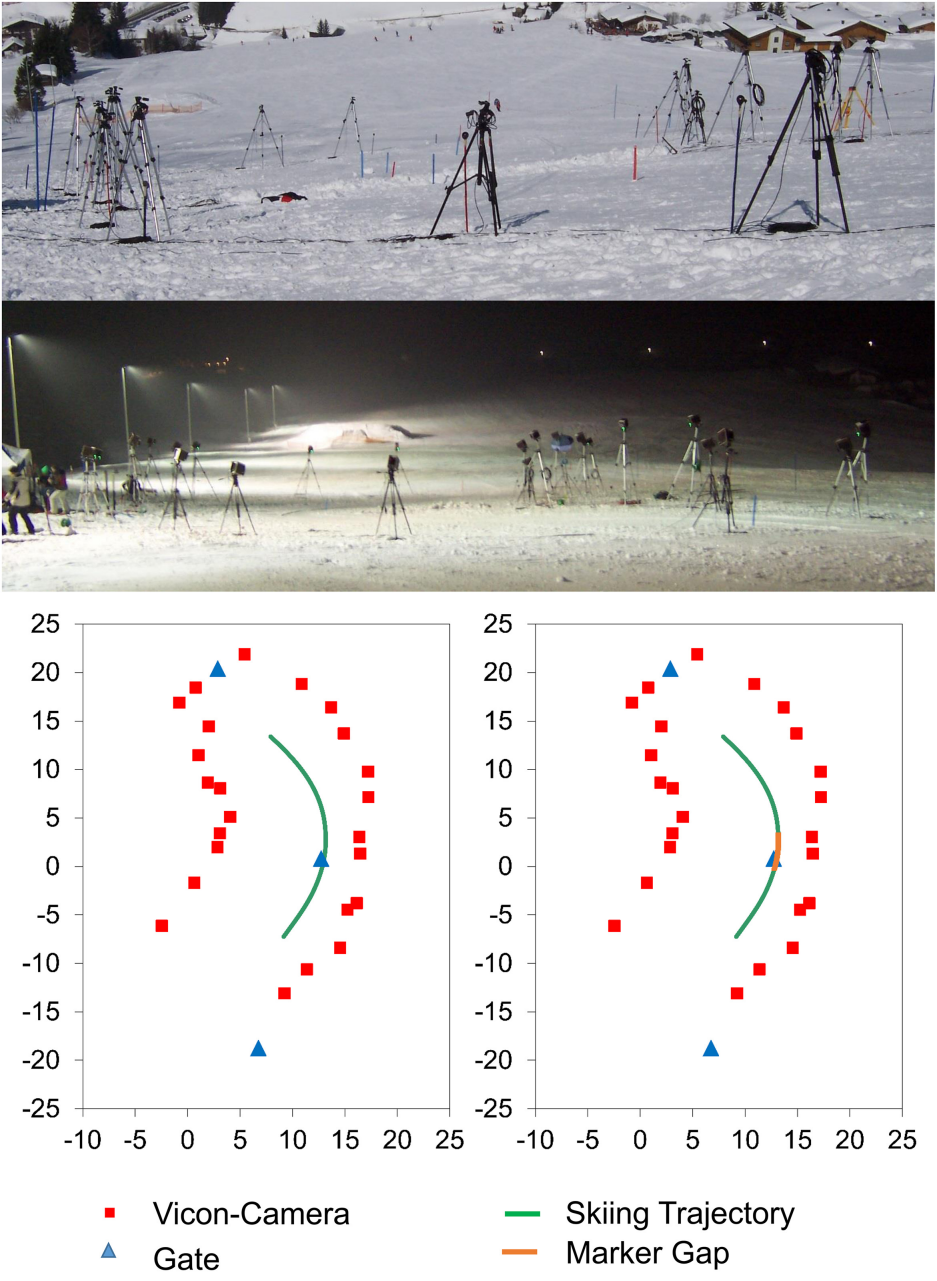
Overview of the measurement-setup. Top: *VICON* camera set-setup. Bottom: schematic drawing of the on-hill measurement setup.

The optoelectronic system consisted of 24 infrared-cameras (*VICON MX 13* & *VICON MX40*, 250 Hz) positioned on 1.8 to 2.6 m high tripods along the analyzed turn (Fig 2). The capture volume covered the space that was needed to perform approximately one ski turn of a pre-defined three gate course (average linear gate distance: 21.2 m; slope inclination 23°). Prior to the aforementioned measurements, the static and dynamic calibrations of the system were performed following the manufacturer’s standard procedure, whereas on the inclined slope the L-frame was aligned perpendicular to gravity with the y-axis representing the direction of the fall line. The camera settings were adapted for the light conditions present on a ski track. Due to the outdoor application of the infrared cameras, the measurement took place at night (i.e. in floodlight conditions). In order to guarantee unrestricted operation of the cameras at temperatures below zero, they were protected by customized heating systems.

### Data evaluation and post processing

For all of the aforementioned measurements and repetitions, markers were labeled, and their positions were reconstructed in 3D using the commercial software *Nexus (VICON Motion Systems Ltd*, *UK)*. All marker distances were directly measured by the use of a caliper/measuring tape (accuracy and precision ≤ 1 mm). The exact distances between the markers of the *VICON* standard wand were provided by the manufacturer (*VICON* Motion Systems Ltd, UK, accuracy and precision ≤ 0.001 mm). For the purpose of error analysis, only unfiltered kinematic data were used. If markers were lost, gaps ≤ 25 frames were interpolated using a *NEXUS* implemented pattern fill, provided that the ambient markers were continuously visible. All other data gaps remained unfilled (for a more detailed description please see S3 Table; unfilled data gaps are recognizable by the value NAN and the red colored background).

### Measures of interest

#### Instrumental errors

For assessing the systematic and random instrumental errors during the static measurement (i.e. 1 trial at rest) and the dynamic measurement (i.e. 1 trial at motion), the optoelectronic system’s *accuracy* for determining inter-marker distances was defined as the arithmetic mean of the absolute differences between the stereophotogrammetrically reconstructed and the manufacturer-specified distances between the three markers of the *VICON* standard wand (= reference values). The system’s *precision* for determining inter-marker distances was defined as the standard deviation of the absolute differences between the reconstructed and the reference values. The system’s maximum instrumental error was defined as the maximum value of the absolute differences between the reconstructed and the reference values within the analyzed static and dynamic trials.

Moreover, the coefficient of variation (CV) was calculated, as was previously recommended by Atkinson and Nevill [20].

#### Skiing-specific errors and soft tissue/suit artifacts

To investigate the magnitude of the occurring total measurement errors including all instrumental and skiing-specific errors (i.e. the effects of higher velocities and substantially obscured markers due to inclined/angulated postures or snow spraying), for two trials of one skier, the distances between the ski boot-fixed, stereophotogrammetrically reconstructed left toe (LTOE) and left ankle (LANK) markers were calculated and compared to the values directly measured by caliper.

In the interest of investigating the magnitude of the total measurement errors that occurred under “typical” skiing conditions (including all instrumental and skiing-specific errors, as well as all soft tissue/suit artifacts), the distances between several skin-fixed, stereophotogrammetrically reconstructed body markers were calculated and were compared to the values directly measured by measuring tape for two trials of one skier.

Subsequently, the absolute differences between the stereophotogrammetrically reconstructed and directly measured inter-marker distances were reported as mean, SD, maximum and CV values.

## Results

### Instrumental errors

The accuracy, precision, maximum instrumental error and CV values for the measurement of the *VICON* standard wand marker distances are presented in Table 1. For both the static and dynamic measurements, the optoelectronic system’s accuracy and precision for determining inter-marker distances were found to be below 0.6 mm and 0.4 mm, respectively. The maximal instrumental error observed was 1.0 mm for the static measurement, and 6.5 mm for the dynamic measurement. For an overview of the individual data points underlying the overall statistics reported in Table 1, refer to S1 and S2 Tables.

**Table 1:** Accuracy, precision, maximum instrumental error and coefficient of variation (CV) values for the measurement of the *VICON* standard wand marker distances a, b and c using an optoelectronic system.

	Accuracy [mm]	Precision [mm]	Maximum Instrumental Error [mm]	CV [-]
**Static measurement** ^a^				
Distance a (390 mm)	0.3	0.2	1.0	0.67
Distance b (260 mm)	0.1	0.1	0.6	1.00
Distance c (130 mm)	0.3	0.1	0.5	0.33
**Dynamic measurement** ^b^				
Distance a (390 mm)	0.6	0.4	3.3	0.67
Distance b (260 mm)	0.4	0.3	5.2	0.75
Distance c (130 mm)	0.3	0.3	6.5	1.00

^a^ Underlying data: 400 frames at rest.

^b^ Underlying data: 905 frames at motion. These are the frames that are required for the skier to ski through the capture volume while carrying and lifting the *VICON* standard wand (average speed: 24.5 km/h).

### Skiing-specific errors and soft tissue/suit artifacts

In general, the measurement errors that occurred (i.e. the absolute differences between the stereophotogrammetrically reconstructed, and directly measured inter-marker distances) became larger when analyzing the distance between the equipment-fixed LTOE—LANK markers of an expert skier at a higher average skiing speed (48.0 km/h), inclined/angulated postures and more substantial snow spraying, than in the aforementioned dynamic measurement (Tables 1 and 2). Under these “typical” skiing conditions, the total measurement errors were found to be 2.3 ± 2.2 mm (Table 2, top).

**Table 2:** Mean, SD, maximum and coefficient of variation (CV) values of the absolute differences between the stereophotogrammetrically reconstructed, and directly measured distances between markers with equipment and skin fixation using an optoelectronic system.

	Mean [mm]	SD [mm]	Maximum [mm]	CV [-]
**Body marker with equipment fixation** ^a^				
LTOE—LANK (288 mm)	2.3	2.2	12.6	0.96
**Body marker with skin fixation** ^b^				
CLAV—STRN (182 mm)	5.8	4.9	22.0	0.85
RASI—LASI (217 mm)	8.3	7.1	34.2	0.86
RPSI—LPSI (119 mm)	3.9	2.9	15.4	0.74
LTHI—LKNE (97mm)	5.4	11.3	57.1	2.09

LTOE: left toe marker; LANK: left ankle marker. CLAV: clavicular marker; STRN: sternum marker; RASI: right anterior pelvic marker; LASI: left anterior pelvic marker; RPSI: right posterior pelvic marker; LPSI: left posterior pelvic marker; LTHI: left thigh marker; LKNE: left knee marker.

^a^ Underlying data: one ski turn performed by an expert level skier (average speed: 48.0 km/h). Please note that due to substantial gaps in marker visibility, for the area between 52.6% and 68.8% of the turn (i.e. frames 243–318), the use of a pattern fill algorithm was not feasible and, therefore, data is not available. Consequently, the data of this area is not included in the corresponding mean, SD, maximum and CV values.

^b^ Underlying data: one entire ski turn performed by an expert level skier (average speed: 48.0 km/h).

Additionally, further skiing-specific artifacts might have been introduced when markers were fixed directly on the skin. In this case, total measurement errors of up to 8.3 ± 7.1 mm were found when, for instance, the distance between the right (RASI) and left (LASI) anterior hip markers was analyzed (Table 2, bottom).

The highest maximum total measurement error (including all instrumental and skiing-specific errors, as well as all soft tissue/suit movement artifacts) was found for the inter-marker distance LTHI—LKNE. For an overview of the individual data points underlying the overall statistics reported in Table 2, refer to S3 Table. Similar magnitudes of total measurement errors as presented in Table 2 were found for a second trial assessed (S3 Table, datasheet 2).

As indicated by footnote “a” in Table 2 and further illustrated in S3 Table, during the turn phase while the skier steers out of the fall line (somewhere between 52.6% and 68.8% of the turn cycle), the visibility of several markers was reduced and occasionally substantial data gaps occurred. In some cases even the use of a pattern fill algorithm was not feasible because ambient markers also were not available. As a result, these gaps remained unfilled.

## Discussion

The main findings of the study were: (1) while at rest or skiing at a low average speed of 24.5 km/h, only minimal snow spraying occurred. In such cases, all markers were fully visible, and the optoelectronic system’s accuracy and precision for determining inter-marker distances on a ski track were found to be below 0.6 mm and 0.4 mm, respectively. (2) Under “typical” skiing conditions (i.e. while skiing at a higher average speed of 48.0 km/h, inclined/angulated postures and moderate snow spraying), additional skiing-specific errors were found to occur for distances between equipment-fixed markers (total measurement errors of 2.3 ± 2.2 mm). (3) For distances between skin-fixed markers such as the anterior hip markers, additional artifacts were observed, resulting in a total measurement of up to 8.3 ± 7.1 mm. (4) Occasionally, the maximum values of the aforementioned total measurement errors were found to be several times higher. (5) Within the turn phase while the skier steered out of the fall line, extensive snow spraying resulted in substantially reduced marker visibility and/or large data gaps.

### Instrumental errors

Generally, the systematic and random instrumental errors of kinematic data collected on a ski track by the use of optoelectronic stereophotogrammetry were found to be comparable with those achievable under laboratory conditions [1–3]. In the absence of substantial velocity effects or obscured markers, the optoelectronic system’s accuracy and precision for determining the distances between stationary or slowly moving markers was found to be below 0.6 mm and 0.4 mm, respectively (Table 1). Based on these findings, we conclude that photogrammetric calibration inaccuracies must have been minimal, and fundamentally, collecting kinematic data on a ski track using optoelectronic stereophotogrammetry is accurate and precise.

### Skiing-specific errors and soft tissue/suit artifacts

In the presence of high skiing speeds, inclined/angulated postures and moderate snow spraying (i.e. “typical skiing conditions”), for distances between equipment-fixed markers, additional skiing-specific errors were found to occur, resulting in total measurement errors of 2.3 ± 2.2 mm (Table 2, top). Thus, compared to the traditional in-field measurement method “video-based 3D kinematics” (marker distance determination accuracy of 9 mm), the observed optoelectronic system’s in-field performance is slightly better [4].

When using markers with skin fixation, for inter-marker distances, additional skiing-specific artifacts were observed (Table 2, bottom); for instance, the resulting total measurement error was found to be 8.3 ± 7.1 mm for the anterior hip markers. Compared to the total measurement error observed for the aforementioned equipment-fixed markers (e.g. LTOE—LANK: 2.3 ± 2.2 mm), this is a difference of 6.0 mm with regard to the mean value. Previous studies assessing the relative movement between skin-fixed markers and the underlying bone under laboratory conditions reported soft tissue artifacts in the range of 10–30 mm [19, 21]. Even if such relative movements were not directly assessed within the current study, the aforementioned total measurement error mean difference between equipment-fixed and skin-fixed markers indicates that soft tissue/suit artifacts are most likely of the same order when collecting kinematic data while skiing.

For most applied research questions in the context of alpine skiing, a measurement system’s accuracy and precision must be below the range of 25–100 mm in order to be able to detect meaningful 3D position- or orientation-related differences [13, 22, 23]. In the current study, the total measurement errors that occurred (including all instrumental and skiing specific errors, as well as all soft tissue/suit artifacts) were found to be markedly lower (Table 2). Thus, we conclude that the accuracy and precision of an optoelectronic system can be considered sufficient for 3D position- or orientation-related analyses on a ski-track, even if a specific research question required a higher accuracy and precision than mentioned above (e.g. below 10 mm).

However, at this point, it must be emphasized that when markers are lost for several frames, and an interpolation based on the patterns of ambient, rigidly connected markers is not feasible (e.g. due to extensive snow spraying), the optoelectronic systems performance might be substantially poorer than observed in the current study. In such a case, an interpolation of large data gaps by unfitting methods might increase the occurring measurement errors drastically.

In this context, it is also worth mentioning that the amount of snow spraying observed in this study represented “moderate” conditions rather than the most extreme case. The skier skied in a controlled low-dynamic recreational skiing style with a relatively low average speed of 48.0 km/h. For more dynamic modes of recreational or competitive alpine skiing, the occurrence of snow spraying is expected to be many times stronger, making a biomechanical analysis of an entire turn almost improbable given the current experimental set-up.

One approach to solving this issue in future experiments might be the use of more sophisticated, cluster-based marker sets. Marker clusters might increase the probability that despite extensive snow spraying, at least some markers remain visible and, therefore, might provide enough input data for a sufficiently accurate reconstruction of missing, ambient markers. Another approach, although possibly irrational, might be the use of additional cameras for snow-spraying-prone sections. However, whether these approaches can help to overcome the snow-spraying-related limitations of optoelectronic systems still needs to be verified by future studies.

### Practical usability of optoelectronic stereophotogrammetry on a ski track

Despite the obvious advantages of using optoelectronic stereophotogrammetry to collect kinematic data on ski track (i.e. the high accuracy and precision for 3D position- or orientation-related analyses and the possibility of at least partially automatic tracking), there are several limitations regarding its practical usability of which potential users should be aware.

First, to define a suitable measurement setup is a tremendously demanding task. In order to guarantee sufficient space coverage by the cameras, and to account for the situation-related limitation of snow spraying, three pilot measurements were conducted for the current study. Moreover, for the construction of the final setup of the main experiment, eight persons worked for approximately one day. Second, due to missing markers (as a result of extensive snow spraying,) data processing time should be expected to be substantially longer than is known under laboratory conditions. Third, in order to guarantee sufficient marker visibility (despite the occurring snow spraying), cameras need to be positioned as high and close to the skiing line as possible (better viewing angles). However, in general, this significantly limits the practical usability of the system (cameras on high tripods), and for applications at higher skiing speeds, such as alpine ski racing, this might limit the comfort and safety of participating subjects when skiing through the measurement area (tight camera corridor). Fourth, due to the static nature of the cameras of the optoelectronic system, a large number of cameras is required to cover the capture volume of one single ski turn, limiting the feasibility of analyzing larger capture volumes (such as skiing sections or entire runs).

Thus, when deciding which measurement system to use for a specific research question/experiment on a ski track, the aforementioned advantages and disadvantages of optoelectronic stereophotogrammetry have to be weighed against each other, and should be compared to those of alternative measurement systems (such as “video-based 3D kinematics”, inertial measurement units, differential global navigation satellite systems [4–8]). In this context, one should also keep in mind that for most skiing-related research questions alternative measurement systems most likely provide both superior practical usability and sufficient accuracy. In some cases, alternative measurement systems may even allow for a more direct way of parameter calculation (e.g. accelerometer for acceleration versus double differentiated position from optoelectronic stereophotogrammetry). The only case of application where the use of optoelectronic stereophotogrammetry seems to be indispensable is if an exact determination of 3D positions/orientations is the highest maxim of the experiment.

### Methodological Considerations

This study aimed to assess the feasibility of collecting kinematic data on an alpine ski track by the use of optoelectronic stereophotogrammetry. It is a pilot study exploring the possibilities and limitations of this system for biomechanical analysis in the specific context of alpine skiing, and might provide important knowledge for further applications in this field. However, the current study does have some limitations that should be kept in mind.

Regarding the criteria of *objectivity*, the current study has two major short-comings. First, the study cannot provide suggestions on how to deal with longer periods of reduced marker visibility/extensive snow spraying. It is obvious that the larger the data gap, the larger the influence of the chosen interpolation method. Second, it is probable that the reported error magnitudes include a certain number of pattern-fill interpolation-induced errors. However, as corresponding interpolations only were made (a) for small gaps (≤ 25 frames, i.e. ≤ 0.1 s at a frame rate of 250Hz), and (b) were based on reliable information provided by an ambient marker fixed on the same harmonically moving segment, their influence on the study outcome might have been marginally small. Third, the current study used one of the simplest marker sets available, the *PLUG-IN-GAIT* model. In this context, it remains unclear whether a pattern fill-based interpolation of missing markers could be further improved by applying other, more sophisticated cluster-based marker sets.

Concerning the criteria of *validity*, the current study is limited by the fact that only one trial/subject per static and dynamic motion task was used for the purpose of error analysis. Obviously this limits the generalization of the study findings (external validity), even if for some of the tasks a second trial revealed similar results. Conversely, as the measurement errors were found to be relatively small, and the skiing-related experiment was performed in the real snow sport environment, the internal validity and ecological validity of the current study can be considered to be sufficiently high. The only limitation to the ecological validity might be the fact that the experiment took place at night (i.e. in floodlight conditions), which is not the most common skiing environment.

Regarding the criteria of *reliability*, the current study lacks in the assessment of the effects of marker placement artifacts. It is known from earlier studies that anatomical landmark misplacements can have substantial effects on the accuracy and precision of stereophotogrammetric systems [24]. On the other hand, there is no reason to expect these effects to be different to those reported for laboratory conditions.

## Conclusions

This study illustrated that on a ski track, the accuracy and precision of optoelectronic systems for determining the distances between stationary or slowly moving passive markers can be considered comparable to the values achievable under in-lab conditions (less than 1 mm). However, when measuring the kinematics of a skier under “typical” skiing conditions (i.e. high speeds, inclined/angulated postures and moderate snow spraying) additional skiing-specific errors and soft tissue/suit artifacts were found to occur. For the distance between the anterior hip markers, for example, total measurement errors of up to 8.3 ± 7.1 mm were observed in the current study. This might be sufficient for the detection of meaningful 3D position- or orientation-related differences in the context of alpine skiing. However, it must be pointed out that as a consequence of extensive snow spraying, partial, but substantial marker obscuration may occur. In addition to a limited practical usability and small-sized capture volumes, this is a serious limitation of using optoelectronic stereophotogrammetry to collect kinematic data on a ski track, and is expected to be even more serious when assessing dynamic modes of recreational or competitive alpine skiing. Whether this issue might be solved by the use of more sophisticated cluster-based marker sets could be subject of future research.

## Supporting Information

S1 Table Static measurement of the *VICON* standard wand marker distances a, b and c (1 trial). AbsDiff: absolute difference between the stereophotogrammetrically reconstructed and the manufacturer-specified distances.
(XLSX)

S2 Table Dynamic measurement of the *VICON* standard wand marker distances a, b and c (1 trial). AbsDiff: absolute difference between the stereophotogrammetrically reconstructed and the manufacturer-specified distances.
(XLSX)

S3 Table Dynamic measurement of selected distances between markers attached to a skier performing turns (2 trials). LTOE: left toe marker; LANK: left ankle marker. CLAV: clavicular marker; STRN: sternum marker; RASI: right anterior pelvic marker; LASI: left anterior pelvic marker; RPSI: right posterior pelvic marker; LPSI: left posterior pelvic marker; LTHI: left thigh marker; LKNE: left knee marker. AbsDiff: absolute difference between the stereophotogrammetrically reconstructed and the manufacturer-specified distances. SD: Standard deviation.
(XLSX)
